# Study of a surface coating present on a Renaissance Piety from the Museum of Ancient Art (Castello Sforzesco, Milan)

**DOI:** 10.1007/s11356-021-16244-9

**Published:** 2021-09-08

**Authors:** Paola Fermo, Mario Colella, Marco Malagodi, Giacomo Fiocco, Michela Albano, Silvia Marchioron, Vittoria Guglielmi, Valeria Comite

**Affiliations:** 1 Dipartimento di Chimica, Università degli Studi di Milano, Via Golgi, 19 Milan, Italy; 2 Dipartimento di Beni Culturali, Università degli Studi di Milano, Via Noto 8, Milan, Italy; 3 Centro studio e conservazione opere d’arte Piccolo Chiostro s.r.l., via C. Procaccini n.4 Fabbrica del Vapore, 20154 Milano, Italy; 4 Arvedi Laboratory of non-Invasive Diagnostics, CISRiC, University of Pavia, Via Bell’Aspa 3, 26100 Cremona, Italy; 5 Department of Musicology and Cultural Heritage, University of Pavia, Corso Garibaldi 178, 26100 Cremona, Italy; 6 Department of Chemistry, Università di Torino, Via Pietro Giuria 7, 10125 Torino, Italy; 7 Department of Physics, Polytechnic of Milan, Piazza Leonardo da Vinci 32, 20133 Milano, Italy

**Keywords:** Marble, Stone degradation, XRF, FT-IR, SEM-EDS, Colorimetric analyses

## Abstract

The surface coating present on a marble Piety dating to the Renaissance period and stored at the Castello Sforzesco-Museum of Ancient Art (Milan, Italy) was studied and chemically characterised. For this purpose, both portable non-invasive (XRF and colorimetric measurements) and micro-invasive techniques (FTIR-ATR and SEM-EDS), have been applied. The statue has been recently submitted to a restoration, since its surface appeared dark and yellowed, before an exhibition at the Louvre Museum and the original appearance of the marble surface recovered thanks to the surface coating removal. Through the analytical characterisation carried out before and after the marble cleaning, the presence of a degradation layer composed by gypsum was evidenced on the stone. The origin of this layer is ascribable to the exposure of the statue to outdoor environment and interaction with atmospheric pollution. The chemical nature of the coating applied at the end of nineteenth century also responsible for the surface alteration was hypothesized.

## Introduction

The issue of conservation and restoration of work of arts stored in museum collections has been of great concern for several decades as broadly attested in the scientific literature and in the field of museum international organisations (Brimblecombe 1992; Blades et al. 2000; ICOM 2008; Lucchi 2018). At this purpose, numerous issues had to be addressed and resolved in the past in order to both restore and prevent further degradation.

**Fig. 1:**
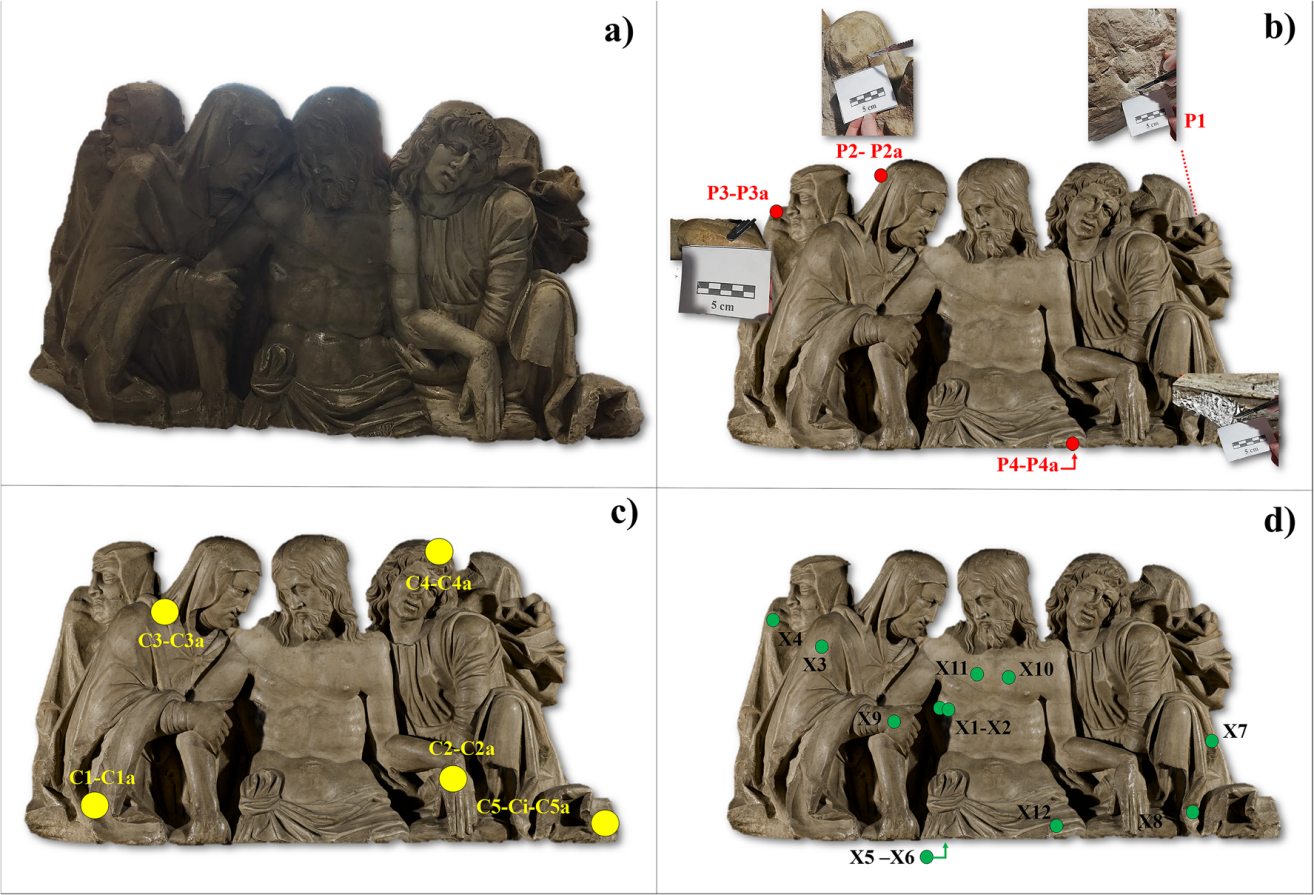
The sculptural group. **a** The three left figures as they appeared before the conservative intervention while the right part already appears in the cleaning phase after a first removal of the coating. **b** The sampling points (P) from where the micro shards before and after the restoration (indicated with a) were withdrawn are displayed in red; **c** The points (C) where colorimetric analysis were carried out before and after the restoration (indicated with a) are displayed in yellow; **d** The XRF measuring points (X) are displayed in green

The present research focuses on the investigation of the surface coatings present on a marble Piety dated to the Italian Renaissance period and stored in the Museum of Ancient Art at the Castello Sforzesco in Milan (Italy) (Fiorio 2014). The monolithic sculptural group, carved in a single block of rock, represents five figures forming a Piety or a Lamentation over the Dead Christ (Fig. 1). This work of art is traditionally attributed to Gasparo da Cairano (Zani 2010), a Renaissance sculptor from the Lombardy or Veneto area (Northern Italy) even if the origin is still uncertain. More in general, according to other critics, the statue could be attributed to a sculptor influenced both by the sculpture of the Duchy of Milan and the artistic workshops of the Republic of Venice (Sgarbi 2006).

It is noteworthy that marble sculptures are often characterised by the presence of surface layers applied as protective coating and restoration treatments (Lanteri et al. 2021) or with the aim to provide an aesthetic finishing.

Furthermore, it is well known that works of arts stored outdoor as well as in museum environments, are subjected to different forms of degradation and alteration (Romuladi et al. 2005, Krupińska et al. 2013, Torralba et al. 2021, Fermo and Comite, 2021) including, for example, the formation of sulphation layers which are due to the reaction of SO_2_ with the marble surface (Krupińska et al. 2013, Comite et al. 2020b, Agelakopoulou et al. 2009).

The marble group object of this study shows traces of, at least, one maintenance intervention due to the application of a protective paint also known among the experts as “*colletta*” (a kind of water-diluted glue most likely made of rabbit glue) that was often applied by museum conservators to protect and enhance the brightness of the marble artworks. In fact, at the turn of the nineteenth and twentieth centuries, it was common to apply organic protective coatings on the statues. These coatings often brought to the formation of dark alterations during time. In the present case, the entire surface of the monolithic group appeared coated with a non-original altered layer which had to be removed.

It was not well documented where the Piety sculptural group was stored in the past but it was probably exposed outdoor for long periods. According to the traditional bibliography, it became part of the Castello Sforzesco collection in 1883, by purchase (Fiorio 2014). During 2020, a restoration was carried out by *Centro studio e conservazione opere d’arte Piccolo Chiostro,* having a wide experience in the restoration of marble and other kinds of materials (Colella, 2015; Colella 2008), since the sculptural group had to be moved at the Louvre Museum (Paris, France) for an important exhibition entitled “The Body and the Soul”, from Donatello to Michelangelo, with 140 Italian Renaissance sculptures exposed, organised in collaboration with the Castello Sforzesco Museum and having as main focus the masterpieces from the second half of the 15th century. During Renaissance, the shapes and movements of the body became of great interest for some of the main artists of this period, starting from Michelangelo. In the exhibition at the Louvre Museum, less famous but no less important artists, such as Gasparo da Cairano, are presented and works of art that are not always accessible because of their usual location in churches, small towns or museum reserves, are exposed. The exhibition was scheduled to be visible from March to June 2021 at the Castello Sforzesco Museum in Milan but unfortunately due to the situation caused by the Covid 19 pandemic this was not possible. Some preliminary investigations (Comite et al. 2020a) have been recently carried out on the Piety in some selected points allowing to formulate hypotheses on the chemical natures of the surface coatings which needed further investigation. The present research was therefore continued with the aim to reconstruct the history of the statue also from the point of view of its conservation.

To disclose the chemical nature and the origin of the residues present on the marble surface different techniques, both non-invasive, such as portable X-Ray Fluorescence (XRF) and portable colorimetric analysis, and micro-invasive such as FTIR-ATR (attenuated total reflection-Fourier infrared spectroscopy) and SEM-EDS (scanning electron microscopy coupled with energy dispersive X-ray spectroscopy) were employed.

## Materials and methods

### The statue

The work of art object of the present research is a sculptural group representing a Piety attributed to Gasparo Cairano (1489?–1517), or to his workshop, dated to the Renaissance period. The dimensions of the statue (Fig. 1) are height 40 cm, width 83.5 cm and depth 25.5 cm. Gasparo Cairano (Zani 2010) was an outstanding artist of the Milanese cultural world at the end of the fifteenth century. He pursued a successful career that soon turned him into the leading exponent of the Renaissance sculpture in Brescia.

The statue was realised employing one of the calcium-silicate dolomitic marbles that became very popular in the Po Valley from the Renaissance. especially in the years when the Duchy of Milan was established, onwards. This was due to the difficulties of importing the traditional Apuan limestones. The Piety marble has a fine grain and a fairly heterogeneous appearance. This pre-Alpine marble is less translucent than Apuan marbles and was probably white marble of Crevola or Crevaldossola (Moro and Negri, 2017), from the name of the place of the quarries (this marble was also known as Palissandro marble).

The statue, belonging to the *Raccolte Artistiche del Castello Sforzesco di Milano* (Fiorio 2014), appeared strongly dark and yellowed (Fig. 1). The restoration coating applied probably at the end of the nineteenth century made in fact the surface yellow and dark due to the progressive absorption of atmospheric dirt. In Fig. 1a, the sculptural group is shown before cleaning (on the left side) and in an intermediate phase after a first removal of the outermost alteration layer (on the right side). The cleaning operation was then completed on the whole surface to bring the statue as it appears in Fig. 1b, c, and d.

### Micro sampling

Some micro shards were taken from the marble surface (Table 1) by means of a scalpel from different areas before and after the cleaning procedure with the aim to highlight the surface coating composition and what was removed from the statue surface. Four points were selected (Fig. 1b), in accordance with the restorer after the permission given by the conservators, and in three of them, the sampling was performed both before and after the cleaning intervention (the samples taken before the restoration are indicated with P1-4 while the samples withdrawn after are indicated with P letter followed by “a” in Fig. 1b). It is important to point out that the samples were taken in the form of powders which were not suitable for preparing cross-sections. Furthermore, it was not possibly to selectively and perfectly separate the outermost layer from the underling surface.

**Table 1:** Samples collected from the statue surface before and after the restoration

	Sampling location on the statue
Sample P1	Sampling performed of the statue basement
Sample P2-P2a	Sampling performed on the back of the Madonna’s head
Sample P3-P3a	Sampling performed on Maddalena’s shoulder
Sample P4-P4a	Sampling performed on the thong of Christ

*a*, after restauration

### Analytical techniques employed

Colorimetric analyses were carried out directly on different areas of the marble surface, before and after the restoration, on the points indicated in Fig. 1c where C1-C5 refer to the measurements before the restoration while the same names followed by “a” letter indicate the measurements on the same points after the cleaning. A Konica Minolta CM 2300d portable spectrophotometer was employed. The measurements refer to the CIE L*a*b* chromaticity diagram and to the Normal recommendation 43/93 (Normal 1933) where L* is luminosity or lightness, which varies from black (value = 0) to white (value = 100); a* ranges from +a* (red) to –a* (green), and b* varies from +b* (yellow) to –b* (blue). Further information on the experimental details is reported elsewhere (Comite et al. 2020a; Guglielmi et al. 2020).

Non-invasive and in-situ EDXRF analyses were performed, on the points indicated in Fig. 1d, with the portable EDXRF spectrometer ELIO (XGLab srl, Milan, Italy) equipped with a low-power X-Ray tube with a Rh anode. The sensitivity range of the spectrometer is 1–40 keV, and it is therefore able to detect elements heavier than Na (Albano et al. 2017; Albano et al. 2020). The measurement parameters were set at time 120 s, tube voltage 40 kV, tube current 40 μA, and acquisition channel 2048. Data were processed using the ELIO 1.6.0.29 software. In Fig. 1d, the measurement points are shown and highlighted in green.

By SEM-EDS semiquantitative analyses were performed on the micro shards (Table 1) without any preliminary preparation using a methodology already set-up (Cappelletti et al. 2005; Guglielmi et al. 2020) by means of a Hitachi TM1000 instrument equipped with an energy dispersive X-ray spectrometer (Oxford Instruments SwiftED).

Infrared spectra were collected on the micro shards in ATR mode by a Nicolet 380 spectrophotometer in the range 4000-500 cm^−1^ at a resolution of 4 cm^−1^.

## Results and discussion

As above mentioned, a coat was applied to the statue (as well evidenced in Fig. 1a) probably at the end of the nineteenth century with the intention of giving the surface a “wet” effect, to close the porosity of the material and to create a sufficient barrier to protect the work during moulding operations. This coat could have produced a darkening of the surface that altered the aesthetic feature of the statue. In this perspective, this layer should be removed. Different methodologies are available for this kind of restoration interventions (Brandi 1963, Matteini and Moles, 1999, Cremonesi 2002, Burnett Grossman et al. 2003), and in the present case, a treatment based on the use of the commercial product Phytagel (Bresciani srl, www.brescianisrl.it/newsite/ita/xprodotto.php?id=6056&hash=ec517bb5644476aa1e99e13273eaf086) was selected and used. This system facilitates the cleaning of the surface thanks to its adhesion and the subsequent tearing, hence removing the dirt layer. Phytagel is a gelling agent, substitute for the well-known agar-agar, commonly used in paper restoration for gentle wet cleaning. It is important pointing out that agar-agar turns yellow whereas Phytagel produces a totally transparent gel. In this way the result shown in Fig. 1b, c was achieved on the statue.

The colorimetric measurements were performed (as indicated in Fig. 1c) on the Madonna’s mantle (C1 and C1a areas before and after restoration respectively), the hand of Christ (C2 and C2a areas, before and after restoration respectively), the Madonna’s dress (C3 and C3a areas, before and after restoration respectively), the angel’s hair (C4 and C4a areas, before and after restoration respectively) and the angel’s dress (in this case, 3 measurements were taken: C5 area before restoration, C5i area during an intermediate stage of the restoration—as represented in Fig. 1a—and C5a area after restoration). Additional areas of the monument were not analysed due to the impossibility of performing accurate measurements because of the non-regular shape of the surface. The values of the colorimetric coordinates are reported in Table 2. An increase in the L* coordinate (luminosity) occurred after the restoration, pointing out a change of the surface shade from dark to a lighter tone. Moreover, L* values after cleaning were more homogeneous thanks to the fact that the dirt was removed and the whiteness of the marble surface appeared. It is important pointing out that one of the requests of the conservators was to try to establish if traces of colours could be present on the surface indicating that the statue was painted.

**Table 2:** Colorimetric coordinates (L*, a*, b*) calculated for the analysed areas before and after restoration

		L*	a*	b*
C1	Before restauration	43.06	4.62	11.83
C1a	After restauration	74.62	1.02	5.52
C2	Before restauration	51.43	4.24	15.29
C2a	After restauration	74.56	2.83	16.93
C3	Before restauration	55.72	2.25	16.23
C3a	After restauration	76.67	4.15	16.98
C4	Before restauration	47.17	4.73	13.47
C4a	After restauration	75.97	3.76	17.34
C5	Before restauration	57.03	2.63	12.69
C5i	Intermediate restauration	73.44	3.96	17.91
C5a	After restauration	75.33	4.15	17.94

The colorimetric coordinate a* increased for areas C3 and C5 while for C1, C2 and C4, a decrease was observed. Since higher values of a* means that the colour is moving towards red, it could be hypothesized, on the base of this observation, to be confirmed with further analyses, that traces of red pigments could be present in C3 and C5 areas. As far as colorimetric parameter b*, it slightly increased for all the analysed areas except for C1 (the mantle of the Madonna) where b* clearly decreased. Considering that negative values of b* are associated with blue colour, the decrease of this coordinate could indicate the presence of traces of a blue shade on the mantle, also in this case to be confirmed with measurements that can highlight the chemical nature of the pigment.

**Fig. 2:**
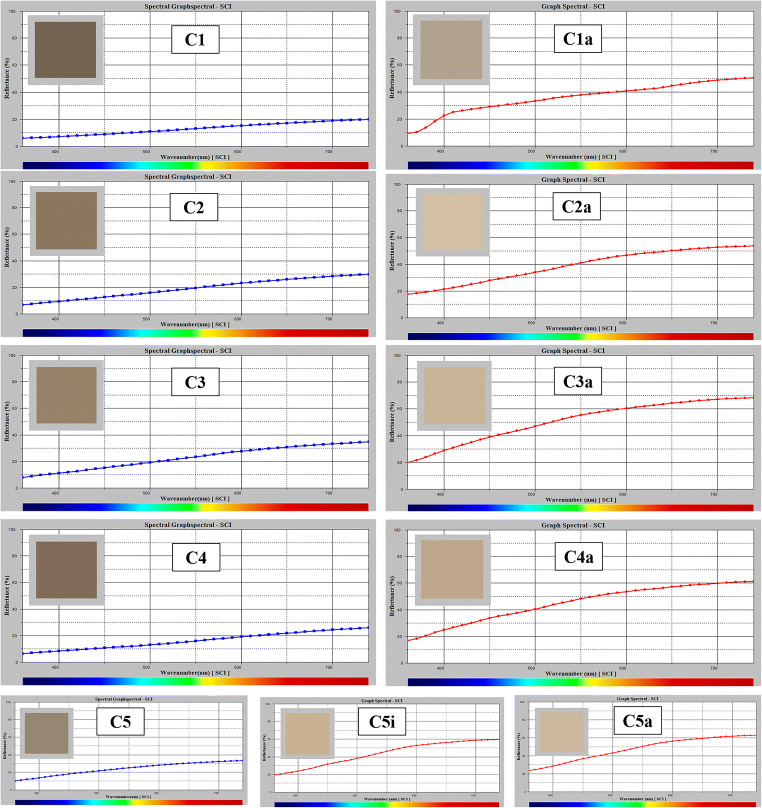
Reflectance spectra of the analysed areas before and after restoration; the squares inside the spectra represent the field of colour

In Fig. 2 for all the investigated areas, the reflectance spectra before and after cleaning are shown together with the colour appearance (in the box inside each figure). All samples show a shift of the colour towards warmer tones after restoration.

For area C1a, an increase in the reflectance in the blue region of the spectrum is clearly observable. In particular  for sample C5a, a clear increase of b* was detected indicating a shift towards the yellow hue.

A non-invasive campaign was accomplished by portable XRF. Data are shown in Tab. 3. The measurements corresponding to areas X4 and X12, as reported in Fig. 1d, were collected respectively in the same region of the P3 and P4 sampling (Fig. 1b); X5 and X6 could be considered as a reference, acquired at the bottom of the sculpture protected by the atmospheric interaction and where no treatments or coatings were expected; X1 and X2 correspond to the cut in Christ’s ribs. The normalised values of the net area counts related to the characteristic elements (Table 3) featured high values of Ca mainly detected together with significative counts of S and Fe. In addition, the signals of Ti, Mn, Zn and Sr were highlighted in few cases.

**Table 3:** Net area count estimation of the peak Kα of the elements detected by XRF on the different areas. Each value was normalized to the mean value—calculated on the whole XRF data set—of the net area counts of the Rh peak (Kα) (Invernizzi et al., 2020). Areas marked with an asterisk (*) were selected as non-treated areas and can be considered as spectral background. Non-detected elements are marked with n.d.

Area	S	Ca	Ti	Mn	Fe	Zn	Sr
X1	5.63	63.92	1.60	n.d.	1.00	1.52	3.50
X2	6.10	58.14	1.05	n.d.	1.05	0.83	3.77
X3	14.44	50.86	0.12	0.25	0.36	0.04	4.17
X4	15.37	63.08	0.17	0.18	0.67	n.d.	4.13
X5*	13.13	43.29	n.d.	n.d.	0.17	n.d.	3.45
X6*	12.50	42.60	n.d.	n.d.	0.18	n.d.	2.76
X7	9.11	34.89	n.d.	n.d.	0.19	n.d.	2.98
X8	9.17	40.26	0.16	n.d.	0.83	n.d.	2.88
X9	10.59	44.35	0.19	n.d.	1.60	0.15	5.81
X10	12.60	46.33	0.12	n.d.	0.16	n.d.	4.69
X11	7.51	58.91	0.74	n.d.	0.73	0.44	3.78
X12	9.32	46.00	0.13	n.d.	0.24	n.d.	3.47

**Fig. 3:**
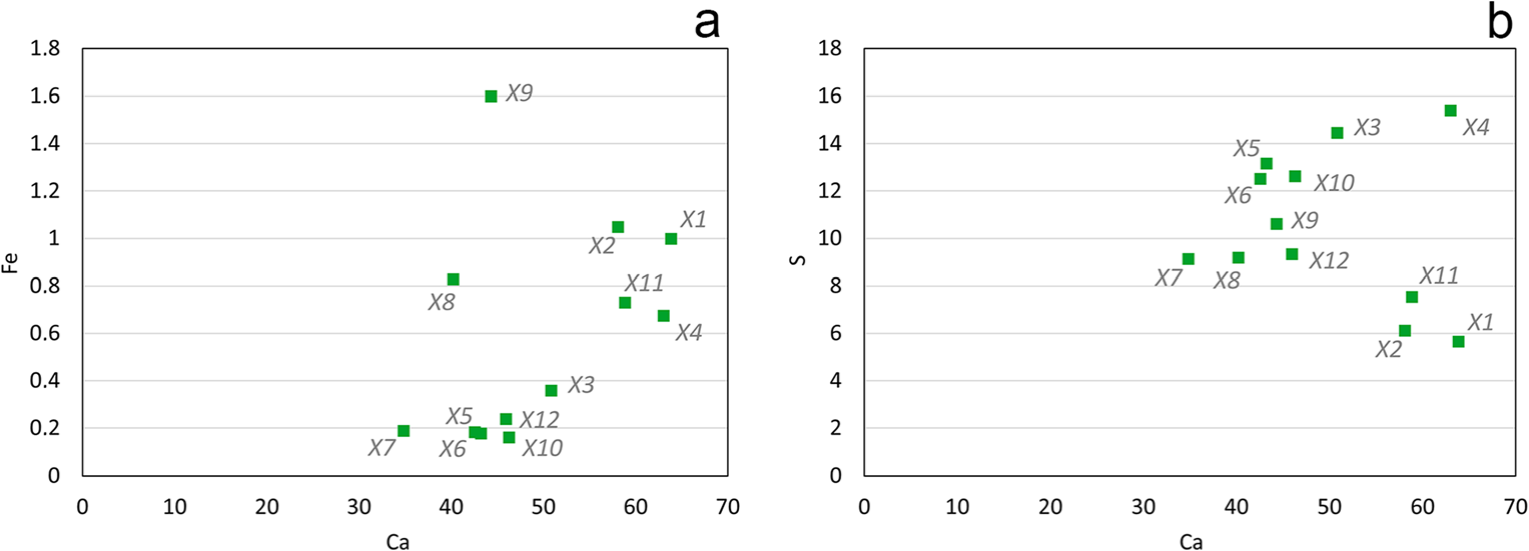
Scatter plot of Ca/Fe (**a**) and of Ca/S (**b**)

If Ca, Mn, Sr and Fe are expected for the original limestone, the even S distribution and the variability of Fe detected by each acquisition are highly informative about the presence of a surface treatment (Invernizzi et al. 2021). The scatter plot of the normalised net area counts Ca/Fe displayed in Fig. 3a, allow us to infer the presence of yellow or red earth pigments. An increase in the Fe counts is recognisable in correspondence to the presumed pigmented areas where reddish or yellowish hue was observed. Those areas, namely X1, X2, X4, X8, X9 and X11, are scattered in a narrow region far from the other group of measurements that are arranged closer to the reference points X5 and X6. The identification of not negligible Fe counts on the X8 analytical spot can be also related to the increasing in the yellow component observed through the colorimetric investigation carried out in the close area C5. Besides, the slight correlation between S and Ca showed in Fig. 3b permitted to hypothesise the presence of gypsum probably employed in a preparation layer or, more likely, an alteration formed by the weathering, as discussed below.

**Fig. 4:**
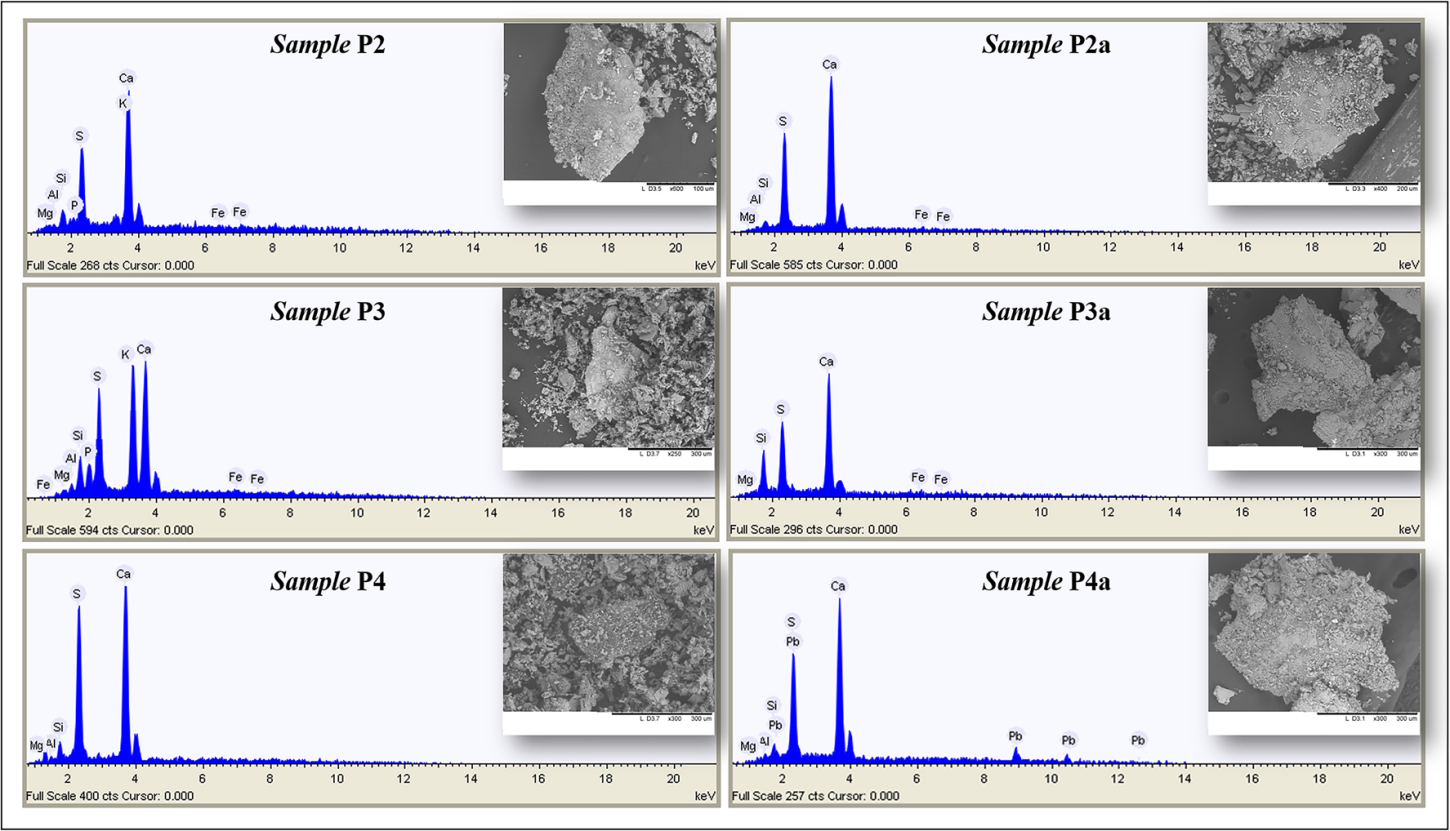
Representative EDS spectra taken before and after restoration on the micro-shards

In order to assess the chemical composition of the micro-shards taken from the surface (Table 1), a characterisation was performed by SEM-EDS both before and after the cleaning procedure. In Table 4 the semi-quantitative data acquired on all the samples are reported. From EDS results, it can be observed that in all the analysed points, an increase of both Ca and S concentrations is evident after the cleaning procedure. Some representative EDS spectra taken before and after restoration are shown in Fig. 4.

**Table 4:** Chemical composition detected by SEM-EDS on some selected areas (and reported as average values) on the fragments taken from the statue surface before and after the restoration (relative standard deviations were in all cases lower than 10%)

**Sample**		**Mg (w%)**	**Al (w%)**	**Si (w%)**	**P** **(w%)**	**S** **(w%)**	**Cl** **(w%)**	**K** **(w%)**	**Ca** **(w%)**	**Fe** **(w%)**	**Pb** **(w%)**
**P1**	Before restauration	0.23	1.30	3.60		36.15			57.28	1.45	
**P2**	Before restauration	0.33	0.40	3.20	0.40	28.07	0.53	2.07	61.83	3.20	-
**P2a**	After restauration	0.30	0.30	2.30	-	30.13	-	-	62.20	4.77	-
**P3**	Before restauration	1.10	0.97	3.87	1.40	25.07	-	30.35	35.65	1.60	-
**P3a**	after restauration	0.90	0.13	2.63	-	29.04	-	-	66.80	0.53	-
**P4**	Before restauration	1.57	1.0	4.70	-	29.77	-	-	62.97	-	-
**P4a**	After restauration	0.27	-	1.47	-	30.73	-	-	63.57	-	3.97

**Fig. 5:**
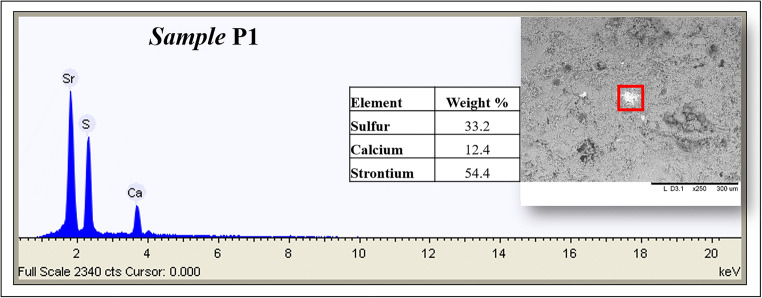
SEM-EDS analysis performed on a particle present on sample P1

Analysis performed on sample P1, where high values of sulphur (36.15%) and calcium (54.4%) were disclosed, confirms that gypsum had been applied in this area to fill a fracture-lacuna, as it appeared from a first visual observation. Moreover, the punctual analysis performed on some particles (Fig. 5) has shown the presence of strontium which is correlated to natural gypsum composition (so in this case not gypsum of neo-formation) indicating a restoration intervention (Barbieri et al. 1976).

**Fig. 6:**
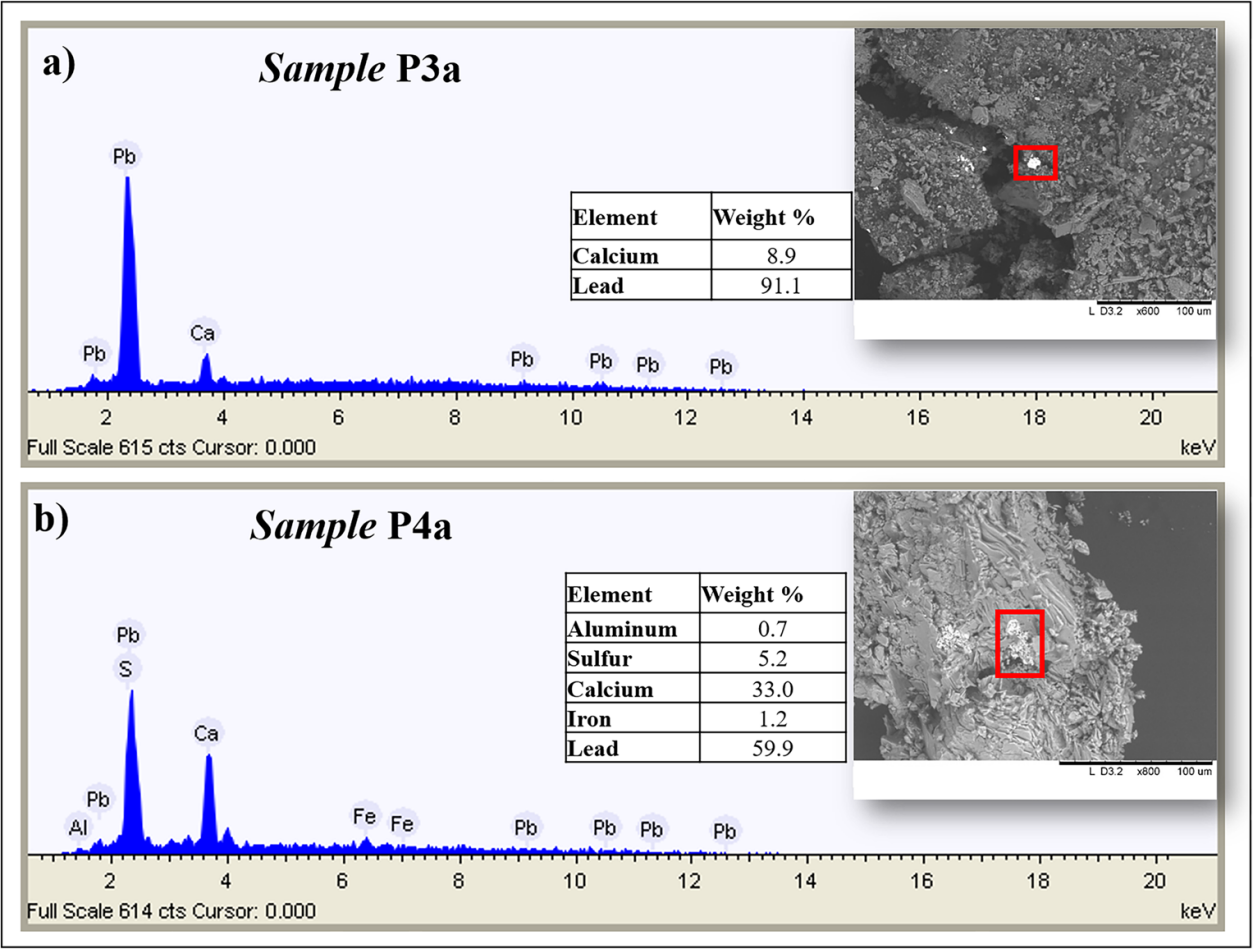
SEM-EDS analysis performed on particles present on the shards taken after restoration. **a** Sample P3a. **b** Sample P4a

The not negligible concentration of lead in P4a on selected areas (Table 4) allowed to hypothesize the use of lead pigments even if further investigation is recommended for the identification of the specific pigment. Furthermore, from analyses carried out on some selected particles (Fig. 6), Pb was found in sample P3a and Pb and Fe in sample P4a. It is worth noting that P3a and P4a correspond to the areas X4 and X12 characterised, on the base of Fe/Ca, by a more red or yellow hue for X4 and a white hue, that could match with the use of a white lead, for X12.

In the case of sample P3, a high quantity of K together with a lower signal of P is observed (Fig. 4 and Table 4). The presence of phosphorus can be attributed to the composition of the “*colletta*” applied to the surface, as described in the introduction. In fact, it was prepared using animal glues produced from rabbit bones.

After the cleaning procedure lower concentrations of Si and Al were detected on the micro shards (Table 4) probably because of the removal of some dust embedded in the surface coating.

Higher concentrations of Ca and S after the restoration (Table 4) have been observed in accordance with what highlighted by XRF analysis. Accordingly to the conservators, the presence of a preparation layer could not be excluded. A further hypothesis suggested the presence of some degradation products like gypsum of neo formation due to the reaction between the marble and atmospheric pollution (Belfiore et al. 2013; Barca et al. 2014; Vidorni et al. 2019), as it will be discussed further on.

**Fig. 7:**
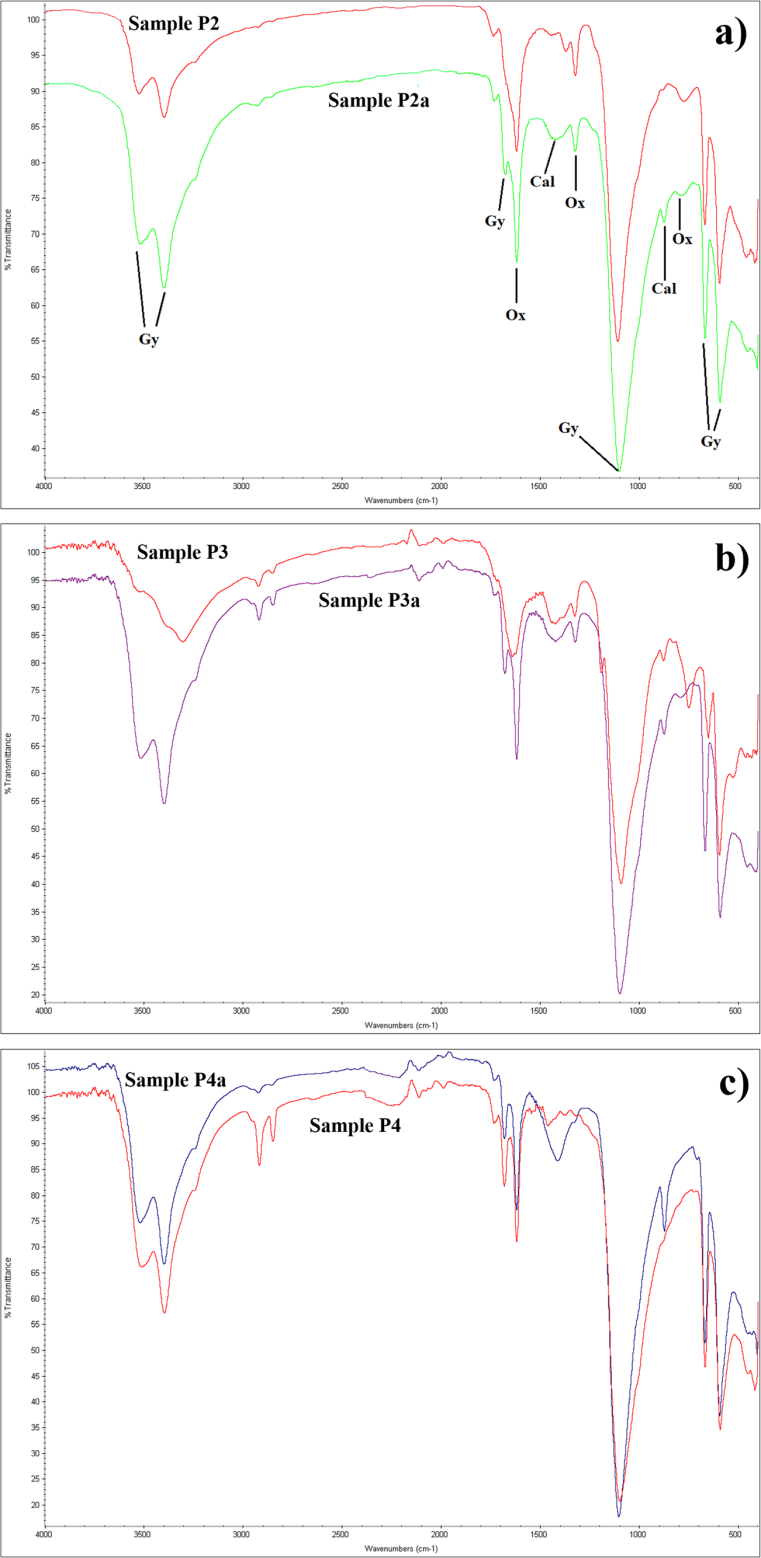
FT-IR spectra acquired on the shards before and after restoration. **a** P2 and P2a. **b** P3 and P3a. **c** P4 and P4a

The widespread presence of Ca and S on the statue surface is also demonstrated by the examination of the three shards. Comparing the FT-IR spectrum acquired before and after the restoration (Fig. 7), gypsum appears more evident after the cleaning as attested by the signals at 3525, 3400, 1627, 1109, 667 and 590 cm^−1^ (Farmer 1974) (signals labelled in Fig. 7a). Together with gypsum also oxalate signals at 1630, 1450, 1380, 1320 and 780 cm^−1^ (Rampazzi 2019) are evident for samples P2, P2a and sample P3 and P3a. Accordingly to the literature oxalate (signals labelled in Fig. 7a) is attributed to the previous restoration interventions or to some biological degradation (metabolism of microorganisms) (Sabbioni and Zappia, 1991; Rampazzi et al. 2004). Furthermore, thanks to the comparison with a spectra data base (https://spectrabase.com/spectrum/4gc0KO4vgqP), it was also verified that no traces of the cleaning product applied on the surface, i.e. Phytagel, remained after its removal.

Another common feature of the three samples is that after cleaning, the signal due to calcium carbonate at about 1420 and 871 cm^−1^ (Farmer 1974) and due to the original limestone is more evident. In the case of sample P4 (Fig. 7c), the signals due to calcite appear only after cleaning (P4a) indicating a greater thickness of the overlying “*colletta*” forming the coating which was not homogenous on the marble surface. In sample P4, the signals at about 2900 cm^−1^ (due to C–H stretching) and at about 1730 cm^−1^ (carbonyl group stretching) together with some quite weak signals at 1530 and 1440 cm^−1^ are indicative of the presence of organic substances due to the proteinaceous nature the “*colletta*”. In fact, the signals at about 1730, 1530 and 1440 cm^−1^ are characteristic of amide I, II and II (Fermo et al. 2020a) present in the amino acid groups of proteins. It is worth noting that this kind of “*colletta*” was prepared, as mentioned before, starting from some animal glue, i.e. a proteinaceous binder. An evident decrease of this signals is observable in sample P4a.

In samples P3a and P4a, stain peaks due to organic substances (at about 1730 cm^−1^ as well as at about 2900 cm^−1^) are still present probably indicating the use of a compound such as oil or wax that was applied to the surface and was not removed with the cleaning (Bonizzoni et al. 2011, Guglielmi et al. 2021). The more plausible hypothesis is that a kind of drying oil was used as a binder and mixed with pigments in order to apply the colour to the stone surfaces (Aguado-Guardiola and Fuster-López, 2017). However, it is known that FT-IR spectroscopy is only indicative of the kind of binder (Bonizzoni et al. 2018, Guglielmi et al. 2021). Nevertheless, the highlighted signals are consistent with the use of an oil, as confirmed by the comparison with some references (https://spectra.chem.ut.ee/paint/binders/). Because of the presence of this oil binder, the hypothesis that a thin preparatory layer of gypsum has been applied on the surface before the colour application, was excluded.

Sample P3 shows two signals at 749 and 650 cm^−1^ which are no longer present after cleaning. The attribution of these signals to a particular compound was not possible. However, as already evidenced, phosphorus and potassium were found on this area by EDS analyses before cleaning but after, no longer appeared (Table 4). The two IR signals could be compatible with the presence of a phosphate on the base of the comparison with literature data (Farmer 1974).

As far as the diffused presence of gypsum on the statue surface, it seems to be much more likely that a sulphation layer has formed during the century on the limestone because of interaction between the surface and atmospheric pollution (the Piety has most likely been placed outdoor for centuries although its history is not well documented).

In fact, it is known that cultural heritage is submitted to a high corrosion risk (De Marco et al. 2017). SO_2_ is responsible, together with other pollutants, of the deterioration of marble statues stored in archaeological museums (Agelakopoulou et al. 2009). It is also well known that in the presence of SO_2_, humidity and aerosol carbonaceous particles, a sulphation process can occur on carbonatic stones. This process corresponds to the chemical transformation of the substrate into gypsum, as well documented in the literature (La Russa et al. 2017; Comite and Fermo, 2018).

Nowadays, SO_2_ air concentration significantly decreased but in the past, large quantities of this pollutant were emitted mainly from coal combustion used as energy and heating sources (Ielpo et al. 2019). The sulphation process, that commonly takes place in outdoor environments, can also bring to different forms of deterioration including black crusts formation (Gulotta et al. 2013; Antonelli et al. 2016; La Russa et al. 2017; Comite and Fermo, 2018; Comite et al. 2020b). However, SO_2_ is not the only component responsible for this degradation and other atmospheric pollutants such as black carbon and heavy metals are also involved (Fermo et al. 2015; Fermo et al. 2020b). As mentioned before, the Piety has been probably exposed outdoor for some period initiating the sulfation phenomenon.

The “*colletta*” applied to the surface of the statue during the nineteenth century, when it became part of the Castello Sforzesco Museum collections, may also have favoured the growth of this layer of gypsum, since this coating certainly did not make the surface of the statue completely impermeable to the permeation of gaseous pollutants and humidity.

It is important pointing out that, in order to protect stone surfaces from such deterioration phenomenon, nowadays, a wide variety of protective coatings are available (Fermo et al. 2014; Germinario et al., 2019; Pargoletti et al. 2019).

In order to investigate more in depth, the origin of the sulphation layer, the possibility of having a cross-section would have allowed to better understand the interaction between gypsum and the underlying stone surface but sampling of a shard of adequate size suitable for this purpose was not permitted in this case by the conservators. Nevertheless, the hypothesis advanced as regards the formation of a degradation layer made of gypsum, seems reliable.

## Conclusions

The application of both non-invasive in situ analyses and micro-destructive investigation permitted to collect information on the chemical composition of the surface coating present on the Renaissance marble sculptural group by Gasparo Cairano.

The comparison of the results gained by the analyses conducted before and after cleaning operations turned out to be a winning strategy. The application of a proteinaceous-based treatment compatible with a “*colletta*”, often employed for conservative purpose at the end of the nineteenth century, has been confirmed thanks to FT-IR analyses. After the removal of this ancient treatment, some colour traces have been highlighted in some areas of the statue, in accordance with a hypothesis advanced by the conservators. In particular, the comparison between XRF and colorimetric results suggested the possible use of yellow or red pigments containing Fe in some areas. It is very likely that these colours have been applied using an oil as a binder, the presence of which became more evident after the removal of the “*colletta*” treatment.

Finally, the removal of the “*colletta*” made it possible, above all, to highlight the presence of an extensive layer of sulphation (gypsum) that may have formed over the centuries because of the interaction between the stone surface and atmospheric pollutants, especially during the period when the statue was exposed outdoor.

All these results have been achieved combining micro-destructive and portable analytical techniques.

The approach applied has also allowed to advance some assumptions on the “conservation history” of this less well known but no less important work of art of the Italian Renaissance period, contributing also to formulate some considerations on its original appearance.

## Data Availability

The datasets used and/or analysed during the current study are available from the corresponding author on reasonable request.
